# Long-Term Follow-Up and Risk of PCOS in Indiana Girls with a History of Premature Adrenarche—A Single Center Experience

**DOI:** 10.3390/children12121609

**Published:** 2025-11-26

**Authors:** Rita Saroufim, Tari Kurman, Erica A. Eugster

**Affiliations:** 1 Pediatric Endocrinology & Diabetology, Indiana University Health, Riley Hospital for Children, Indianapolis, IN 46202, USA; eeugster@iu.edu; 2 Department of Pediatrics, Indiana University School of Medicine, Indianapolis, IN 46202, USA

**Keywords:** premature adrenarche, Polycystic Ovary Syndrome (PCOS), adolescent health, diabetes, pre-diabetes, dyslipidemia

## Abstract

**Background/Objectives**: The aim of this study was to investigate rates of self-reported Polycystic Ovary Syndrome (PCOS), metabolic abnormalities and age of menarche in girls with a history of Premature Adrenarche (PA) and the association of baseline characteristics with these outcomes. **Methods**: A retrospective chart review of girls with PA seen between 2005 and 2015 was conducted. Eligible patients (or their mothers in girls <18 years of age) were surveyed by telephone to query whether they had developed PCOS, diabetes, pre-diabetes, hypertension or dyslipidemia as well as their age of menarche. Associations of baseline characteristics with PCOS or metabolic abnormalities were analyzed. **Results**: A total of 359 patients with PA were identified, of whom 118 completed a telephone survey. At baseline, the average age of girls with PA was 6.7 ± 1.4 years, of whom 55% were White. At follow-up, the average age of girls was 17 ± 3.5 years. Among the respondents, 19.5% reported a diagnosis of PCOS, 5.1% reported diabetes, 8.5% pre-diabetes, 7.6% hypertension and 6% hyperlipidemia. Major risk factors for PCOS included a maternal history of PCOS (*p* = 0.005, OR 4.1) and increased BMI at baseline (*p* = 0.03, OR 1.69). Additionally, girls with history of PA had an earlier age of menarche compared to their mothers (11.3 ± 1.6 years vs. 11.9 ± 1.5 years, *p* = 0.002). **Conclusions**: Our results suggest that maternal history of PCOS poses the greatest risk for future development of self-reported PCOS in girls with a history of PA followed by increased BMI. Prospective studies using objective diagnostic criteria are needed to corroborate these findings, as our outcomes were based on self-reported data.

## 1. Introduction

Premature Adrenarche (PA) is characterized by clinical evidence of androgens prior to the age of 8 years in girls and 9 years in boys. It is manifested by any combination of pubic and/or axillary hair, acne and adult body odor [1]. PA occurs far more often in girls than in boys at a ratio of 10:1 [2]. Since pathological causes of increased androgen exposure can have a similar presentation, PA is a diagnosis of exclusion. Although PA is commonly encountered in clinical practice, it remains poorly understood. PA is believed to reflect altered timing and/or amplitude of the physiological release of the androgen precursors dehydroepiandrosterone (DHEA) and dehydroepiandrosterone sulfate (DHEA-S) by the zona reticularis of the adrenal glands [2]. The subsequent conversion of DHEA to androstenedione and testosterone in the gonads and peripheral tissues leads to the hallmark clinical signs as described above [2].

Established risk factors for PA include obesity, minority ethnicity, positive family history and having been born small for gestational age (SGA) [3]. In girls born SGA, PA is considered to exist within a cascade of metabolic derangements that have in common a state of insulin resistance. While initially regarded as a benign variant of normal development, longitudinal studies of girls with PA performed primarily in Spain have reported an increased incidence of metabolic syndrome, type 2 diabetes (T2DM) and Polycystic Ovary Syndrome (PCOS) developing during adolescence in these patients [4]. Moreover, some investigators have described PA as a forerunner for PCOS and metabolic syndrome, in line with the Barker (fetal origins of adult diseases) hypothesis [5]. However, whether these results can be generalized to a population of girls with PA from the American Midwest is unknown. The aim of this study was to investigate rates of self-reported PCOS, metabolic abnormalities and age of menarche in girls with a history of PA who were seen in a large tertiary center in Indiana and determine whether baseline characteristics were associated with those outcomes.

## 2. Methods

A retrospective chart review of patients seen at Riley Hospital for Children between 2005 and 2015 was conducted. Since the specific diagnosis code for PA was not introduced until 2015, the diagnosis codes (ICD-9) used in the search criteria for the specified time frame included ICD-9 255.3 (other cortico-adrenal overactivity) and ICD-9 259.1 (precocious sexual development and puberty, not elsewhere classified). Although there was no formal ICD term, providers consistently documented the diagnosis of PA in their notes based on established clinical features and exclusion of alternative pathologic causes. Only patients whose charts explicitly documented a clinical diagnosis of PA were included. Girls who presented with pubertal disorders or pathologic conditions like non-classic congenital adrenal hyperplasia, adrenal or ovarian tumors or exogenous androgen exposure were excluded. Demographics, birth and maternal history, biochemical studies, bone age and clinic visit information were collected (timepoint A). Eligible patients or their mothers (in girls under 18 years of age) were surveyed by telephone to query whether they were diagnosed with PCOS, pre-diabetes, diabetes, hypertension or dyslipidemia, and their age of menarche (timepoint B). Telephone surveys were conducted between January 2022 and August 2023. The difference between the two timepoints was deliberately chosen so that the girls with a history of PA would be old enough to have potentially developed the outcomes of interest. To ensure consistency and minimize interviewer bias, all telephone surveys were conducted by two of the authors who strictly adhered to a unified script (included in Supplementary Materials). Associations of baseline characteristics with PCOS and T2DM were analyzed using logistic regression. Statistical analysis was performed using SPSS version 28. A *p*-value of <0.05 was considered statistically significant. Multivariate logistic regression was performed using the enter-method in SPSS, in which all selected variables are entered simultaneously. Variables were included in the model if they demonstrated statistical significance in univariate analysis at *p* < 0.05 or were considered clinically relevant based on prior literature. This study was approved by the Institutional Review Board at Indiana University Health under exempt status (IRB #2011678092). Verbal informed consent was obtained from patients or their mothers to participate in the telephone survey.

## 3. Results

A total of 2,050 patient charts were reviewed. Of those, 1691 patients were excluded (116 were boys, 1234 had other puberty disorders, and 341 charts were incomplete). A total of 359 girls with PA were identified (timepoint A). At follow-up (timepoint B), 118 (33%) participants completed the telephone survey (6 declined participation and 235 were unreachable). Of those, 50 participants (~42%) were ≥18 years old and completed the telephone survey themselves. Only the patients who completed the survey were included in the analysis. Included patients were similar in baseline characteristics to those who did not complete the survey, with the exception of a lower proportion of African American patients (28% vs. 39%, *p* = 0.01) and a slightly higher bone age/chronological age ratio (BA/CA) (1.2 vs. 1.14, *p* = 0.04) (Table 1).

At timepoint A, the average age of girls at diagnosis of PA was 6.7 ± 1.4 years with the majority being White (55%). The most common chief complaint was early pubic hair development (77%) followed by adult body odor (13%). Most patients (70%) were born at term and 20% were born SGA. Their average BMI on presentation was 19.8 ± 4.1 kg/m^2^ with a z-score of 1.3 ± 1.1. The average (BA/CA) was 1.2 ± 0.2 and average DHEA-S was 71.8 ± 38.6 mcg/dL. The average reported maternal age of menarche was 11.9 ± 1.5 years. Details of baseline characteristics of both included and non-participant patients are presented in Table 1.

The average duration for follow-up (from timepoint A to timepoint B) was 10.4 years ± 3.7 years. The average age of the participants at follow-up was 17 years ± 3.5 years. Of those who completed the survey, 19.5% (23/118) reported having received a diagnosis of PCOS by their primary care provider, 5.1% (6/118) type 2 diabetes, 8.5% (10/118) pre-diabetes, 7.6% (9/118) hypertension, and 6% hyperlipidemia (7/118). Of those who did not have a formal diagnosis of PCOS, 17% reported having irregular menses and/or clinical signs of hyperandrogenism (acne and hirsutism). The reported average age of menarche was 11.3 ± 1.6 years which was younger than that of their mothers 11.9 ± 1.5 years, *p* = 0.002.

Univariate logistic regression analysis revealed that a maternal history of PCOS and increased BMI at baseline were associated with increased risk of self-reported PCOS (*p* = 0.005, OR 4.1; *p* = 0.03, OR 1.7, respectively). African American ethnicity was associated with a decreased risk of self-reported PCOS (*p* = 0.007, OR 0.1). Age at diagnosis of PA, pubic hair tanner stage, having been born SGA, and androgen levels were not associated with increased risk of self-reported PCOS (Table 2). On multivariate analysis, maternal history of PCOS, BMI and ethnicity remained statistically significant (*p* = 0.04, OR 3.4; *p* = 0.01, OR 2.3; *p* = 0.01, OR 0.13, respectively).

Regarding the risk of diabetes, having increased BMI at baseline was associated with an increased risk of diabetes (*p* = 0.03, OR 4.4) on univariate analysis, but no other baseline characteristics were statistically significant (Table 2). Due to small sample size, a multivariate analysis could not be performed.

## 4. Discussion

PA has been regarded as a possible risk factor for PCOS since the 1990s when several studies demonstrated signs of functional ovarian hyperandrogenism [6], increased insulin resistance and dyslipidemia in adolescent girls with a history of this common variation of development [4,5]. The etiology behind PA remains unclear but low birth weight, prematurity and minority ethnicity are considered risk factors [7]. In our cohort, 20% of the girls with PA were born SGA, around one-fourth were born premature, and ~40% were of minority ethnicity (African American and Hispanic). Prior studies have reported higher rates of overweight and obesity in children with PA, and our cohort demonstrated a similar pattern, with girls exhibiting elevated BMI z-scores relative to age- and sex-specific norms [7,8] (Table 1). Among our patients, pubic hair (77%) was the most reported main chief complaint followed by adult body odor (13%) and axillary hair (7%). DHEA-S is the main hormone involved in the pathophysiology of PA [7], and our results show a moderate positive correlation (r = 0.388, *p* < 0.01) between DHEA-S levels and Tanner stage of pubic hair development, indicating that higher DHEA-S levels are associated with more advanced pubic hair Tanner stages.

The age of menarche in girls with PA was previously reported to be similar to that in the general population [1]. However our study found an earlier age of menarche in girls with PA compared to their mothers and the general population [9] which is in line with what has been reported by a recent study [10].

PCOS is estimated to affect approximately 8–13% of women of reproductive age of various ethnicities, varying by diagnostic criteria [11]. Our study revealed a higher self-reported incidence of ~20% in girls with a history of PA which aligns with previous longitudinal studies from Spain and Brazil. For instance, studies from Spain have reported PCOS incidences ranging from 15% to 40% [5,8] while a Brazilian cohort reported an incidence of 41% [12] depending on diagnostic criteria and follow-up duration. In our study, a maternal history of PCOS posed the highest risk factor for self-reported PCOS in girls with PA (OR = 4.1), while a higher BMI increased the risk by 1.7 times. Conversely, our study showed that African American girls with a history of PA had lower odds of self-reported PCOS despite minority ethnicity being a known risk factor for the condition. However, they were also underrepresented at follow-up, which introduces selection bias. Because this group had a lower observed risk, their reduced participation may have contributed to an overestimation of overall PCOS incidence and should temper interpretation of African American ethnicity as a protective factor. Moreover, the model’s relatively low sensitivity on multivariate analysis suggests that additional factors may be important in predicting PCOS in this population. Age at PA diagnosis was a marginally significant risk factor (*p* = 0.052) that could have reached significance with a larger sample size. We also found that obesity increased the risk for self-reported type 2 diabetes four-fold. While obesity alone is a known risk factor for insulin resistance and diabetes, having a history of PA may compound this risk, underscoring the need for targeted interventions.

Our study has several limitations including its retrospective nature which may introduce recall bias and incomplete data, as well as the self-reported nature of the study outcomes.

First, the relatively low follow-up rate may have introduced selection bias. Only a subset of the original cohort completed the follow-up survey, and responders included a lower proportion of African American girls compared to non-responders. Because African American participants in our cohort were found to have a lower risk of self-reported PCOS, their underrepresentation could have led to an overestimation of the overall incidence of PCOS in the study group. Conversely, the reported rates in our cohort may well have under-represented the true prevalence of PCOS, as 17% of girls in our study without a formal diagnosis reported suggestive symptoms.

Second, diagnoses were based on self-reported information, which is subject to recall and reporting biases. Both over- and under-reporting are possible. However, prior literature suggests that underreporting is more common for chronic and reproductive conditions such as PCOS, as many affected women remain undiagnosed or unaware of their condition [13]. This limitation could therefore have resulted in an underestimation of the true prevalence of PCOS, diabetes, or prediabetes in our cohort. Future studies incorporating standardized diagnostic assessments or linkage to medical records may provide more accurate estimates.

In addition, maternal PCOS history was extracted from the initial clinic visit as documented in the medical chart and, when possible, was confirmed again during the follow-up telephone survey. Despite this verification, some degree of recall inaccuracy cannot be excluded. The same limitation applies to maternal age at menarche, which was also obtained from clinic records and re-confirmed at follow-up. Although prior studies demonstrate that maternal recall of age at menarche is remarkably reproducible, some inaccuracy is still possible [14]. Given the modest follow-up rate and reliance on self-reported diagnoses, the incidence rates of PCOS and metabolic conditions reported here should be interpreted with caution.

Our sample size is relatively large compared to other studies although the follow-up rate (33%) was lower than desired. However previous literature has shown that response rates in survey-based research commonly range between 30 and 50%, and a 33% response rate is considered acceptable for studies with hard-to-reach populations or limited contact options [15,16]. Furthermore, to our knowledge ours is the first study to investigate long-term self-reported outcomes of PA in a cohort of girls from the United States.

## 5. Conclusions

In conclusion, our study provides preliminary insights into the long-term outcomes of girls with PA in the American Midwest. In this cohort, self-reported data suggest a higher incidence of PCOS and earlier menarche compared to the general population, with maternal history of PCOS and increased BMI at baseline emerging as significant risk factors. Although these findings should be interpreted in the context of the study’s retrospective design, low follow-up rate, and reliance on self-reported diagnoses, they highlight important reproductive and metabolic trends that warrant attention in clinical practice. These results support the importance of anticipatory guidance and ongoing follow-up for girls with a history of PA, particularly in those with additional risk factors (like elevated BMI or a maternal history of PCOS) and emphasize the need for larger prospective studies to confirm and expand on these observations.

## Figures and Tables

**Table 1:** Comparison of Baseline Characteristics Between Included and Non-participant Girls with Premature Adrenarche (timepoint A).

Characteristics	Included Patients, n = 118 (Mean ± SD or n/N, %)	Non Participants, n = 241 (Mean ± SD or n/N, %)	*p* Value
Age (years)	6.7 ± 1.4 (n = 118)	6.7 ± 1.5 (n = 241)	0.6
Ethnicity - White - African American - Hispanic - Biracial	55% (58/105) 39% (41/105) 1.0% (1/105) 1.9% (2/105)	70% (147/209) 28.1% (59/209) 1.0% (2/209) 0.5% (1/209)	0.01
Chief Complaint - Pubic hair - Body odor - Axillary hair - Acne	77% (91/118) 13% (15/118) 7% (8/118) 0.8% (1/118)	73% (175/241) 19% (45/241) 5% (11/241) 0.8% (2/241)	0.6
Gestational age - Term - Premature - Post-term	70% (74/105) 27% (28/105) 3% (3/105)	75.3% (122/162) 22% (35/162) 3% (5/162)	0.6
% SGA ^a^	20% (19/96)	19% (28/145)	0.9
Pubic hair Tanner Stage - Tanner I - Tanner II - Tanner III - Tanner IV	16% (19/118) 54% (64/118) 25% (30/118) 4% (5/118)	13% (32/241) 59% (141/241) 25% (61/241) 3% (7/241)	0.7
Acanthosis Nigricans	8% (9/112)	8% (19/232)	0.9
BMI (kg/m^2^) - BMI z-score	19.8 ± 4.1 (n = 118) 1.3 ± 1.1 (n = 118)	20.1 ± 6.7 (n = 241) 1.3 ± 1.0 (n = 241)	0.7 0.9
BA/CA ^b^	1.2 ± 0.2 (n = 97)	1.14 ± 0.2 (n = 195)	0.04
DHEA-S (mcg/dL)	71.8 ± 38.6 (n = 82)	81.1 ± 46.3 (n = 164)	0.1
Total Testosterone (ng/dL)	7.57 ± 13.3 (n = 75)	7.72 ± 10.6 (n = 152)	0.9
Maternal age of Menarche (years)	11.9 ± 1.5 (n = 79)	11.86 ± 1.6 (n = 133)	0.8
Family history - PCOS ^c^ - Metabolic abnormalities (T2DM ^d^ &/or hyperlipidemia &/or hypertension)	15% (12/82) 44% (52/118)	18% (24/133) 41% (58/140)	0.6 0.7

Abbreviations: ^a^ SGA: small for gestational age; ^b^ BA/CA: Bone Age/Chronological Age; ^c^ PCOS: Polycystic Ovary Syndrome; ^d^ T2DM: Type 2 Diabetes Mellitus.

**Table 2:** Association of baseline characteristics with risk of self-reported PCOS ^a^ and type 2 diabetes.

Outcome	Variable	Univariate OR (95% CI)	Univariate *p* Value	Multivariate OR (95% CI)	Multivariate *p* Value
PCOS ^a^	Maternal PCOS ^a^	4.10 (1.53–11.37)	0.005	3.43 (1.00–11.71)	0.04
	Ethnicity—African American vs. White	0.12 (0.03–0.56)	0.007	0.13 (0.03–0.67)	0.01
	Age at PA ^b^ diagnosis	1.44 (0.99–2.08)	0.052	-	-
	BMI z-scores at baseline	1.69 (1.04–2.75)	0.03	2.35 (1.21–4.59)	0.01
	SGA ^c^	0.94 (0.27–3.21)	0.92	-	-
	DHEA-S ^d^	1.00 (0.98–1.01)	0.73	-	-
	Androstenedione	1.03 (0.99–1.06)	0.12	-	-
	17-OHP ^e^	1.01 (0.99–1.01)	0.07		
	Tanner Staging			-	-
	Tanner II	0.75 (0.06–9.27)	0.82		
	Tanner III	1.22 (0.12–11.80)	0.86		
	Tanner IV	0.61 (0.05–6.99)	0.69		

Diabetes	Ethnicity—African American vs. White	1.40 (0.67–2.95)	0.37	-	-
	Age at PA ^b^ diagnosis	0.96 (0.57–1.62)	0.89	-	-
	BMI z-scores at baseline	4.41 (1.17–16.55)	0.03	-	-
	SGA ^c^	1.25 (0.13–11.37)	0.84	-	-
	DHEA-S ^d^	1.01 (0.98–1.03)	0.47	-	-
	Androstenedione	0.90 (0.93–1.05)	0.81	-	-
	17-OHP ^e^	0.99 (0.97–1.02)	0.82	-	-

Abbreviations: ^a^ PCOS: Polycystic Ovary Syndrome; ^b^ PA: Premature Adrenarche; ^c^ SGA: small for gestational age; ^d^ DHEA-S: dehydroepiandrosterone sulfate; ^e^ 17-OHP: 17-hydroxyprogesterone.

## Data Availability

The data presented in this study are available on request from the corresponding author (RS) due to containing information that could compromise the privacy of research participants.

## Supplementary Materials

The following supporting information can be downloaded at: https://www.mdpi.com/article/10.3390/children12121609/s1, Survey script.
