# Supplementary material for: Long-Term Follow-Up and Risk of PCOS in Indiana Girls with a History of Premature Adrenarche—A Single Center Experience
Source: Children (Basel). 2025 Nov 26;12(12):1609. doi: 10.3390/children12121609 (PMC12731558; doi:10.3390/children12121609)
Supplement: Supplementary file 1 [file children-12-01609-s001.zip › children-3976667-supplementary.pdf]

Informed consent script:

Participant ID#

Hi, this is [Dr. Rita Saroufim / Tari Kurman] from Riley Hospital for Children. We're doing a follow-up study on girls who were seen here for premature adrenarche, including you/your daughter, about ___ years ago. I'm calling to ask if you would be willing to answer a few questions about health history since that visit.

The survey takes about 5–10 minutes. Your participation is voluntary, your answers will be kept confidential, and nothing you say will affect any medical care. You may skip any question or stop at any time.

We will use the information only in summary form, without names, to help doctors better understand the long-term health of girls with premature adrenarche. There are no direct benefits or risks other than the time it takes to answer the questions.

If you have any questions, you can reach me at 317-944-3181.

Do you agree to participate in this study?

[Record "Yes" or "No."]

Parent version

Indiana University School of Medicine

Department of Pediatric Endocrinology

Project title: The risk for metabolic syndrome in Indiana girls with a history of premature adrenarche

Patient Study ID number:

a. What is your child's current weight? ____ Kg/lbs

b. What is your child's current height? ___inch/cm

c. Menstrual History:

   1. At what age did your child have her first period? ____

   2. Are the periods regular? If no, please describe:

Yes ___

No: __________

___N/A (or not achieved menarche yet)

d. Have your child been diagnosed with any of the following?

   1. PCOS (Polycystic Ovary Syndrome)? Yes No _ _N/A

   2. Pre-diabetes? ___Yes ___ No

   3. Diabetes? ___Yes __No

   4. High cholesterol or triglycerides? __Yes ___ No

   5. Hypertension or is having high blood pressure? ___Yes ___ No

e. Does your child have acne or increased male pattern hair (eg on face, chest, abdomen)?

__Yes (indicate): _________

__No:

f. Does your child take any medications? If yes, can you please list them?

____ Yes: __________

____ No

g. Has the child's mom been diagnosed with PCOS or diabetes?

h. At what age did the child's mom have her first period?

i. Does any of the child's siblings or parents have diabetes or high lipid? If yes, please specify.

__ Yes: ____

___No

j. If we are able to bring your child in to our clinic to check some hormone levels at no cost to you, may we contact about that at a later date? ___Yes ___ No

Study patient version:

Indiana University School of Medicine

Department of Pediatric Endocrinology

Project title: The risk for metabolic syndrome in Indiana girls with a history of premature adrenarche

Patient Study ID number:

a. What is your current weight? ____ Kg/lbs

b. What is your current height? ___inch/cm

c. Menstrual History:

    3. At what age did you have your first period? ____

    4. Are the periods regular? If no, please describe:

Yes ___

No: __________

___N/A (or not achieved menarche yet)

d. Have you been diagnosed with any of the following?

    6. PCOS (Polycystic Ovary Syndrome)? Yes No _ _N/A

    7. Pre-diabetes? ___Yes ___ No

    8. Diabetes? ___Yes __No

    9. High cholesterol or triglycerides? __Yes ___ No

    10. Hypertension or is having high blood pressure? ___Yes ___ No

e. Do you have acne or increased male pattern hair (eg on face, chest, abdomen)?

__Yes (indicate): _________

__No:

f. Do you take any medications? If yes, can you please list them?

____ Yes: __________

____ No

g. Has the your mom been diagnosed with PCOS or diabetes?

h. At what age did your mom have her first period?

i. Does any of your siblings or parents have diabetes or high lipid? If yes, please specify.

__ Yes: ____ ____

___No

j. If we are able to bring you in to our clinic to check some hormone levels at no cost to you, may we contact about that at a later date? ___Yes ___ No
